# Attitudes and practices related to intrauterine devices for nulliparous women among Chinese health care providers

**DOI:** 10.1186/s12978-019-0678-9

**Published:** 2019-01-31

**Authors:** Ziliang Wang, Chaohua Lou, Longmei Jin, Maohua Miao, Xiaowen Tu, Hong Liang, Yan Cheng, Wei Yuan

**Affiliations:** 1 NHC Key Lab. of Reproduction Regulation (Shanghai Institute of Planned Parenthood Research), Fudan University, Shanghai, 200237 China; 2 Minhang Maternal and Child Health Care Hospital, Shanghai, 201102 China; 3 Family Planning NSW, Sydney, 2131 Australia

**Keywords:** Intrauterine device, Nulliparous women, Health care provider, Attitudes, Practices, China

## Abstract

**Background:**

Unintended pregnancy and induced abortion among young (unmarried or nulliparous) women have become serious social and health issues in China, which are thought to partly result from low use of long-acting reversible contraception, such as intrauterine devices (IUDs). Considering that providers may play an especially important role in increasing use of this particular method, we investigated Chinese health care providers’ attitudes and practices regarding IUDs use for nulliparous women, and further examine the potential factors and reasons contributing to their attitudes and practices.

**Methods:**

We recruited health care providers at two Chinese national academic conferences and surveyed them about knowledge of IUDs, willingness to recommend IUDs to nulliparous women, and their related practices. Modified Poisson regression was used to examine the potential factors related to their attitudes and practices.

**Results:**

Approximately a half of respondents reported unwillingness to recommend IUDs to nulliparous women. Providers with more misperceptions about the safety and contraindications were more likely to be unwilling to recommend IUDs to nulliparous women. Moreover, 71.0% of respondents rarely or never recommended or provided IUDs to nulliparous women. Providers’ unwillingness to recommend IUDs to nulliparous women was associated with their infrequent practices (aPR=1.43; 95% CI: 1.04, 1.96). In addition to concerns about the side effects or complications, traditional sexual concept against premarital sex was often cited as a reason by Chinese health providers for their negative attitudes towards IUDs use for nulliparous women, a large proportion of whom are unmarried.

**Conclusions:**

Our findings suggest that negative attitudes and infrequent practices regarding IUDs use for nulliparous women are common in Chinese health care providers. Moreover, misperceptions about IUDs, as well as traditional sexual concept, contribute to Chinese health care providers’ negative attitudes towards IUDs use for nulliparous women, and may further reduce their provision. Educational interventions are needed to improve providers’ accurate knowledge of IUDs and fill the gap between traditional sexual concept and young women’s needs of contraceptive services.

## Plain English summary

Unintended pregnancy and induced abortion among young (unmarried or nulliparous) women have become serious social and health issues in China. Although Intrauterine devices (IUDs) are highly effective and recommended as a first-line contraceptive method for all women, IUDs use in unmarried women or nulliparas is markedly low in China. Considering that providers may play an especially important role in increasing use of this particular method, we conducted a survey among health care providers at two Chinese national academic conferences to understand their attitudes and practices regarding IUDs use for nulliparous women, and further examine the potential factors and reasons contributing to their attitudes and practices. We found negative attitudes and infrequent practices regarding IUDs use for nulliparous women were common among our respondents, and providers with more misperceptions about IUDs were more likely to be unwilling to recommend IUDs to nulliparous women. In addition, traditional sexual concept against premarital sex was often cited as a reason by Chinese health providers for their negative attitudes towards IUDs use for nulliparous women, a large proportion of whom are unmarried. This study highlights the importance of educational interventions to improve providers’ accurate knowledge of IUDs and fill the gap between traditional sexual concept and young women’s needs for contraceptive services.

## Background

Unintended pregnancy and induced abortion pose important public health challenge across the world, as well as in China. According to the Chinese “Health and Family Planning Statistics Yearbook” (2016) [1], about 10 million induced abortions are performed annually in China, approximately accounting for 25% of the total number in the world. Moreover, the occurrence of induced abortions in young or nulliparous women is relatively high, while 47.5% of women seeking abortion were under the age of 25 and 49.7% were nulliparous [2]. A comprehensive review showed that among unmarried women, 12-32% had a previous pregnancy, most of which were unintended, and the prevalence of induced abortion among unmarried women was over 20% [3].

High unintended pregnancy rates are thought to partly result from low use of long-acting reversible contraception [4, 5], such as intrauterine devices (IUDs), which are user-independent and exhibit higher compliance than short-acting methods, with typical use failure rates of <1% [6–8]. Use of IUDs has great potential to allow young women to progress from age-of sexual initiation to age-of –readiness for parenthood without an unintended pregnancy. IUDs have been the most prevalent method of contraception in China since the 1950s [9]. Among the worldwide IUDs users, 92 million IUDs users (60%) are in China [10]. However, while IUDs is the major contraceptive method for parous women in China, use in unmarried women or nulliparas is markedly low (0%-3%) [11].

Although the safety of IUDs in nulliparas has been well illustrated, with very low rates of complications such as pelvic inflammatory disease (PID) (1.6 cases per 1000 woman-years) or ectopic pregnancy (0-0.5 per 1000 women-years) [12–15], and IUDs were recommended as a first-line contraceptive method among all women, including adolescents and nulliparous women [16, 17], confusions regarding contraindications and patient eligibility for IUDs use persist. Several studies examining health care providers’ knowledge about IUDs found that many providers lacked important information on IUDs safety and eligibility [18–21]. Moreover, providers may play an especially important role in increasing use of this particular method among young women as study shows that accurate information about IUDs can be difficult to find on the internet compared with information on other methods [22]. Lack of knowledge would affect providers’ willingness and practice to recommend or provide this method, and further present barriers to increasing IUDs use among young women [20, 23, 24].

To our knowledge, no previous studies have assessed Chinese health care providers’ knowledge, attitudes and practices regarding IUDs use for nulliparous women. In addition, findings from most previous studies were based on western culture and social background, different from which, however, Chinese traditional culture emphasizes virtue of chastity and sees premarital sex as a taboo [25]. Although dramatic social and economic development in the last four decades in China has led to changed attitudes towards sex [3, 26], we hypothesized that traditional culture environment would still be important factors influencing providers’ attitudes and practices regarding contraceptive use for young women. This study was designed to examine Chinese health care providers’ attitudes and practices related to IUDs use for nulliparous women, which might be crucial determinants of popularizing IUDs. In order to guide intervention development, we also sought to explore potential factors and reasons contributing to their attitudes and practices, including providers’ knowledge of IUDs, their underlying values, and other related demographic and occupational characteristics.

## Methods

Data for this study came from a cross-sectional anonymous survey with a convenience sample of health care providers. We recruited participants from the Chinese Academic Conference on Obstetrics and Gynecology (July 2-5, 2015) and the 13^th^ National Academic Conference on Women’s Health Care (September 17-20, 2015). A self-administrated questionnaire was distributed in sessions on contraception topics to query health care providers on demographic characteristics (sex, age, educational level), occupational characteristics (professional title, working years, provision of contraceptive services, receipt of training in IUDs, work setting and location, on-site availability of IUDs), knowledge of IUDs, attitudes towards IUDs use for nulliparous women, and their practices to provide or recommend IUDs to nulliparous women. Questionnaire was designed taking previous studies as a reference [20, 23, 27, 28], pilot-tested with family planning service providers and epidemiologists, and revised based on the feedback from the pilot.

We assessed providers’ knowledge of IUDs, including their awareness of safety, effectiveness, complications, and contraindications. For safety, respondents were asked if they considered IUDs to be a safe contraceptive method (very safe, safe, unsafe, very unsafe, and unsure). Responses of unsafe, very unsafe, or unsure were defined as misperceptions about the safety of IUDs [20]. Knowledge about the effectiveness of IUDs was assessed by asking about how many women out of 100 would experience an unintended pregnancy (contraceptive failure) with one year of typical use (<1%, 1-5%, 6-10%, and >10%) [7, 27]. Typical use failure rate <1% was defined as correct answer, while 1-5% and >5% were defined as slightly and strongly underestimation, respectively [7, 29]. Knowledge about the complications was assessed with a 5-quesiton test, by asking if there was an association between IUDs and infertility, PID, sexually transmitted infections (STIs), cancer or ectopic pregnancy [27]. Knowledge about the contraindications was assessed with a 6-quesiton test, by asking if respondents considered adolescents, being unmarried, being married nulliparous, having a history of STIs within past 2 years, having a history of PID within past 5 years, or in a nonmonogamous relationship as contraindications of IUDs [23, 28]. The number of wrong answers out of these questions was calculated, respectively, as the knowledge score about the complications and contradictions of IUDs. Respondents with knowledge scores ≥ median were categorized as having more misperceptions.

Our current analyses examined two main outcomes of interest for IUDs: health care providers’ attitudes towards IUDs use for nulliparous women, and their practices related to IUDs for nulliparous women. With respect to attitudes towards IUDs use, respondents were asked if they were very willing, willing, unwilling, or very unwilling to recommend IUDs to nulliparous women. Responses of unwilling or very unwilling were grouped as negative attitudes and compared with the reference category of respondents who were “willing” or “very willing” to recommend IUDs to nulliparous women. Additionally, we examined reasons for negative attitudes towards IUDs for nulliparous women. Respondents who indicated unwillingness to recommend IUDs to nulliparous women were further asked to indicate reasons. Potential reasons included concerns about difficulties in insertion, concerns about side effects or complications, concerns about risk of medical dispute, concerns about follow-up process and removal of IUDs after insertion, concerns about expense, perceptions that nulliparous women wouldn’t accept IUDs, perceptions that IUDs use seems against traditional sexual concept because most nulliparous women are unmarried, or others. Respondents could select more than one reason.

To measure frequency of provision or recommendation practices, respondents who had been providing contraceptive services (including counselling or treatment) were asked how often they provided or recommended IUDs to nulliparous women in the past year: often, sometimes, rarely, or never. Respondents who indicated that they rarely or never provided or recommended IUDs to nulliparous women were classified as infrequent providers and were compared with the reference category of those who often or sometimes provided or recommended IUDs to nulliparous women. We also collected information on respondents’ practices to provide or recommend IUDs to parous women, and compared the infrequency of practices for nulliparous women with that for parous women.

We examined possible factors related to negative attitudes towards IUDs for nulliparous women, including health care providers’ demographic characteristics, occupational characteristics, and knowledge of IUDs mentioned above. We additionally included health care providers’ attitudes towards IUDs when examining possible factors related to their infrequent provision or recommendation of IUDs to nulliparous women. In univariate analyses, Chi-squared analysis or Fisher’s Exact Test was used to assess statistical significance. Modified Poisson regression with robust error variance using simultaneous entry was further applied to compute multivariable-adjusted prevalence ratios (PRs) and 95% confidence intervals (CIs) for possible related factors, including health care providers’ demographic and occupational characteristics with *p* value<0.2 in univariate analyses, and variables of knowledge about the safety, effectiveness, complications and contraindications of IUDs. All statistical analyses were performed using Stata15.0 (Stata Corp., LP, College Station, TX).

## Results

A total of 103 health care providers completed the survey and returned the questionnaires, yielding a response rate of 76.3%; Ninety-one percent of them were obstetrics and gynecology physicians, while a few participants were family planning practitioners or public health providers. The demographic and descriptive characteristics of the participants are presented in Table 1. The majority had educational level of college or higher (91.1%). Most participants had medium (35.3%) or senior (43.1%) professional title, and worked in district (42.1%) or city hospital (30.4%). The majority had been providing contraceptive services (85.1%) or had received training in IUDs (79.6%).

**Table 1:** Characteristics of Health Care Providers Surveyed and Key Variables

Characteristics	N (%)
Sex	
Male	1 (1.0)
Female	98 (99.0)
Age	40.4±8.3
Professional title	
Junior	22 (21.6)
Medium	36 (35.3)
Senior	44 (43.1)
Educational level	
Graduated from technical school or less	9 (8.9)
College graduate	67 (66.3)
Postgraduate or higher	25 (24.8)
Working years	
≤10 years	32 (31.7)
>10 years	69 (68.3)
Setting	
Community hospital	17 (16.7)
District hospital	43 (42.1)
City hospital	31 (30.4)
Others	11 (10.8)
Location	
Urban	80 (80.8)
Suburban	19 (19.2)
Providing contraceptive services	
Yes	86 (85.1)
No	15 (14.9)
On-site availability of IUDs	
Yes	92 (91.1)
No	9 (8.9)
Receipt of training in IUDs	
Yes	82 (79.6)
No	21 (20.4)
Knowledge about the safety of IUDs	
Very safe or safe	92 (90.2)
Very unsafe or unsafe or unsure	10 (9.8)
Knowledge about the effectiveness of IUDs	
Correctly or slightly underestimated	84 (84.0)
Strongly underestimated	13 (16.0)
Score of Knowledge^a^ about the complications of IUDs	
<3 (median)	44 (46.3)
≥3	51 (53.7)
Score of Knowledge^a^ about the contraindications of IUDs	
<5 (median)	37 (41.1)
≥5	53 (58.9)
Willingness to recommend IUDs to nulliparous women	
Very willing or willing	48 (51.6)
Very unwilling or unwilling	45 (48.4)
Provision or Recommendation of IUDs to nulliparous women	
Often or sometimes	20 (29.0)
Rarely or never	49 (71.0)
Provision or Recommendation of IUDs to parous women	
Often or sometimes	67 (91.8)
Rarely or never	49 (8.2)

^a^Knowledge about the complications and contradictions were assessed with a 5-quesiton test and a 6-question test, respectively. The number of wrong answers out of these questions was calculated as the knowledge score

Most respondents (90.2%) perceived IUDs as very safe or safe. One fifth (21.0%) correctly reported the effectiveness of IUDs, while 63.0% slightly underestimated the effectiveness. However, misperceptions about the complications and contraindications of IUDs were common, with median (interquartile range) knowledge score of 3 (2, 4) and 5 (4, 5), respectively (Table 1).

Approximately a half of respondents reported that they were very unwilling or unwilling to recommend IUDs to nulliparous women (Table 1). Among them, we examined reasons cited for the negative attitudes towards IUDs use for nulliparous women (Table 2). The most commonly cited reason for unwillingness to recommend IUDs was “nulliparous women themselves wouldn’t accept IUDs” (57.8%). Additionally, concerns about side effects or complications were often cited (48.9%). Notably, a total of 37.8% of respondents cited “IUDs use seems against traditional sexual conception because most nulliparous women are unmarried” as a reason. In multivariable analysis, the prevalence of unwillingness to recommend IUDs to nulliparous women varied by educational level, and misperceptions about the safety and contraindications of IUDs (Table 3). Health care providers with higher educational level were less likely to be unwilling to recommend IUDs to nulliparous women (aPR=0.43; 95% CI: 0.26, 0.71), while having more misperceptions about the safety (aPR=1.66; 95% CI: 1.05, 2.61) and contraindications (aPR=3.46; 95% CI: 1.76, 6.81) was associated with health care providers’ unwillingness to recommend IUDs to nulliparous women.

**Table 2:** Reasons for Unwillingness to Recommend IUDs to Nulliparous Women

Reasons for being very unwilling or unwilling to recommend IUDs	N (%)
Concerns about difficulties in insertion	8 (17.8)
Concerns about side effects or complications	22 (48.9)
Concerns about risk of medical dispute	7 (15.6)
Concerns about the follow-up process and removal of IUDs after insertion	13 (28.9)
Concerns about the expense	1 (2.2)
Nulloparous women wouldn’t accept IUDs	26 (57.8)
IUDs use seems against traditional sexual concept because most nulliparas are unmarried	17 (37.8)
Others	0 (0.0)

**Table 3:** Adjusted PRs of Unwillingness to Recommend IUDs to Nulliparous Women

Characteristics (Yes vs. No)	aPR^a^	*P*
Professional title ≥ medium	0.92 (0.51, 1.65)	0.770
Educational level ≥ college graduate	0.43 (0.26, 0.71)	0.001
Providing contraceptive services	0.92 (0.51, 1.65)	0.778
Receipt of training in IUDs	0.78 (0.39, 1.57)	0.487
Perceiving IUDs as very unsafe or unsafe	1.66 (1.05, 2.61)	0.030
Strongly underestimating the effectiveness of IUDs	1.21 (0.69, 2.11)	0.511
Having more misperceptions about the complications of IUDs^b^	1.09 (0.67, 1.78)	0.719
Having more misperceptions about the contraindications of IUDs^b^	3.46 (1.76, 6.81)	<0.001

^a^Adjusted for all the variables in this table.

^b^Knowledge scores ≥median.

Among respondents who had been providing contraceptive services, the prevalence of infrequent provision or recommendation of IUDs to nulliparous women was much higher than that to parous women (71.0% vs. 8.2%, *p*<0.001). In multivariable analysis, the prevalence of infrequent provision or recommendation of IUDs to nulliparous women varied by work setting and health providers’ willingness to recommend IUDs to nulliparous women (Table 4). Providers who worked in district hospital had a higher prevalence of infrequent provision or recommendation than those working in other settings (aPR=1.37; 95% CI: 1.03, 1.81). Additionally, respondents reporting unwillingness to recommend IUDs to nulliparous women had a much higher prevalence of infrequent provision or recommendation than those reporting willingness (aPR=1.43; 95% CI: 1.04, 1.96).

**Table 4:** Adjusted PRs of Infrequent Provision or Recommendation of IUDs to Nulliparous Women

Characteristics (Yes vs. No)	aPR^a^	P
Educational level ≥ college graduate	1.67 (0.57, 4.84)	0.346
Working in district hospital	1.37 (1.03, 1.81)	0.028
Perceiving IUDs as very unsafe or unsafe	0.60 (0.32, 1.11)	0.104
Strongly underestimating the effectiveness of IUDs	0.81 (0.50, 1.31)	0.392
Having more misperceptions about the complications of IUDs	0.94 (0.70, 1.25)	0.664
Having more misperceptions about the contraindications of IUDs	1.21 (0.86, 1.69)	0.275
Unwillingness to Recommend IUDs to Nulliparous Women	1.43 (1.04, 1.96)	0.027

^a^Adjusted for all the variables in this table.

## Discussion

Although most health care providers considered IUDs as a safe contraceptive method, approximately a half of respondents reported unwillingness to recommend IUDs to nulliparous women. The prevalence of unwillingness was even higher among certain populations, health care providers with lower educational level, or with more misperceptions about the safety and contraindications. Moreover, 71.0% of respondents who had been providing contraceptive services rarely or never recommended or provided IUDs to nulliparous women. Those who worked in district hospital had a higher prevalence of infrequent recommendation or provision. In addition, providers’ unwillingness was associated with their infrequent recommendation or provision of IUDs to nulliparous women.

Previous studies reported that health care providers’ overall knowledge of IUDs was positively associated with their likelihood to recommend IUDs [21, 30]. In United States, due to the backlash against IUDs, mainly caused by complications from the Dalkon Shield in the 1970s [31], there are many safety misperceptions that persist with modern-day IUDs [32]. Common misperceptions include perceived risks of PID, infertility and ectopic pregnancy with IUDs use [18, 19, 23, 33]. This has greatly contributed to the perception that IUDs are not appropriate for young or nulliparous women. Similarly in the current study, when we further explored different aspects of knowledge, misperceptions about the contraindications and safety of IUDs were most strongly associated with providers’ unwillingness to recommend IUDs to nulliparous women.

However, when we examined reasons for unwillingness to recommend IUDs to nulliparous women, the most commonly cited reason was that nulliparous women themselves wouldn’t accept IUDs (57.8%). This is similar to previous studies that indicated health care providers were heavily influenced by patient preferences [20, 24]. However, it should be noted that providers may play an especially important role in increasing patients’ awareness of this particular method as accurate information about IUDs can be difficult to find on the internet compared with information on other methods [22]. This would produce a cycle in which providers who are unwilling to recommend IUDs to nulliparous women do not educate their patients on knowledge of IUDs, the patients do not ask for services regarding IUDs since they haven’t receive enough information from providers, and, as a result, providers perceive that the patient is not interested in IUDs and would not recommend IUDs to them [24].

Another important finding was that the following cited reason for providers’ unwillingness, except for concerns about side effects or complications of IUDs (48.9%), was “IUDs use for nulliparous women seems against traditional sexual concept because most of them are unmarried” (37.8%). Unlike western values on which most previous studies were based, Chinese traditional culture emphasizes asceticism, with a strict code regarding premarital sexual behavior [34]. In the past four decades, China has experienced dramatic social changes associated with rapid economy growth and reform. Attitudes towards sex, marriage and family have changed, with the growing prevalence rate of premarital sex in young people [3, 26]. However, the health care services did not adapt well to the evolving change. Providers reported negative attitudes towards IUDs for nulliparous women partly because they perceived that IUDs use among young women might be a sign of regular or frequent sexual activity before marriage, which seems inappropriate under Chinese traditional environment. Thus, given the fact that the number of sexually active young people in China is growing [26], it is important to fill the gap between traditional stigma of premarital sex and young unmarried women’s unmet needs for effective contraception in China.

It is not surprising that providers’ willingness to recommend IUDs to nulliparous women was associated with their practices. Compared with parous women, the prevalence of infrequent provision or recommendation of IUDs to nulliparous women was much higher in our study. It was consistent with previous studies reporting that IUDs was the major contraceptive method for parous women in China, while the use in unmarried women or nulliparas was markedly low [11]. Current evidence showed that complications of IUDs (PID, perforation, expulsion, ectopic pregnancy, and fertility) are all of very low incidence, and differ little between nulliparous and parous women [32, 35]. In addition, IUDs insertion has not been shown to be more difficult in nulliparous women compared with parous women [36], and IUDs continuation and satisfaction rates are high among nulliparous women [37–39]. Unlike the low use of IUDs in western countries, IUDs have been the most prevalent method of contraception among parous women in China [9, 10, 40], which built the capacity of health care providers in providing related services and enabled good availability of IUDs in China. Therefore, increasing IUDs use among nulliparous women might be promising in China if effective interventions in terms of improving the accurate knowledge of IUDs and developing an appropriate attitude towards premarital sex could be conducted, and this begins in the education of health care providers.

The main strength of this study is that we first examined health care providers’ attitudes and practices regarding IUDs use for nulliparous women in China, with its unique traditional culture and prevalent IUDs use among parous women. We further tried to explore the underlying values and concerns behind their negative attitudes or infrequent practices. In addition, we also examined some potential characteristics related to providers’ attitudes and practices regarding IUDs use for nulliparous women, and the results suggested educational intervention should be highlighted in certain populations of providers, such as those with lower educational level, or working in district hospital.

Despite these strengths, our study should be interpreted in light of certain limitations. The survey was administered at national conferences and questionnaires were distributed in sessions related to contraception. However, the convenience sampling method limited the generalizability of our results. The sample size was small and thus hindered our ability to further analyze results for different types of clinical and nonclinical providers. Statistically insignificant findings may be due in part to the sample size. In addition, the providers who participated in our survey and returned the completed questionnaire were likely biased in favor of those interested in IUDs and thus might have better knowledge than nonparticipants.

## Conclusions

Our findings suggest that negative attitudes and infrequent practices regarding IUDs use for nulliparous women are common in Chinese health care providers. Moreover, providers’ misperceptions of the safety and contraindications of IUDs are associated with their negative attitudes towards IUDs use for nulliparous women, and may further reduce their provision of related services. It is also important to note that Chinese health care providers are influenced heavily by traditional sexual concept when they provide contraceptive services to unmarried women. Educational interventions are needed to improve providers’ accurate knowledge of IUDs and fill the gap between traditional sexual concept and young women’s needs of contraceptive services.

## Abbreviations

CIs: Confidence intervals
IUDs: Intrauterine devices
PID: Pelvic inflammatory disease
PRs: Prevalence ratios
STIs: Sexually transmitted infections
